# Technology for Young Adults with Stroke: An Australian Environmental Scan

**DOI:** 10.3390/ijerph21091254

**Published:** 2024-09-21

**Authors:** Dinah Amoah, Sarah Prior, Matthew Schmidt, Carey Mather, Marie-Louise Bird

**Affiliations:** 1 School of Health Sciences, University of Tasmania, Launceston 7250, Australia; matthew.schmidt@utas.edu.au (M.S.); marie-louise.bird@utas.edu.au (M.-L.B.); 2 Tasmanian School of Medicine, University of Tasmania, Burnie 7320, Australia; sarah.prior@utas.edu.au; 3 School of Nursing, University of Tasmania, Launceston 7250, Australia; carey.mather@utas.edu.au; 4 Department of Physical Therapy, University of British Columbia, Vancouver, BC V6T 1Z4, Canada

**Keywords:** environmental scan, apps, interactive games, digital platform, social media, young stroke

## Abstract

Technology has the potential to address the unique needs of young stroke survivors. Despite this, little is known about the technological resources available to support young adults with stroke. This study aimed to identify and compile available technological resources that cater to the specific needs of young adults (18–30 years) with stroke in Australia. An environmental scan was conducted from December 2023 to March 2024. Sources included websites, app stores, rehabilitation centres, hospitals, organisations, technology developers, and healthcare professionals. Of the 114 resources identified, 11% were for re-training limb movement, 40% for speech rehabilitation, 20% for medication reminders, and 29% were social media posts offering peer mentoring and support. Most limb movement (75%) and medication reminder (87%) apps were free. However, most speech therapy apps (78%) had associated costs. Social media posts were hosted on Facebook (64%), Instagram (21%), TikTok (9%), YouTube (3%), and other websites (3%). Forty-six percent of the social media posts targeting young stroke survivors did not specify the age group. These resources were identified as available to young people with stroke. Although the resources found focused on young stroke survivors, it was difficult to ascertain the specific age group that was being targeted.

## 1. Introduction

Stroke is a global public health concern and is one of the leading causes of mortality and morbidity, according to the 2019 Global Burden of Diseases study [1,2]. Notably, the incidence of stroke among people aged under 70 years has increased by 15% from 1990–2019 [3]. This trend is reflected in Australia, where it is estimated that 24% of stroke cases in 2020 were among individuals younger than 54 years old [4].

The economic repercussions of stroke among younger individuals are substantial as they grapple with lasting disabilities during their most productive years and beyond [5]. For those aged under 30 years, the impact of stroke can be even more profound than for those who are older. This impact and the repercussions have essential implications, as young adults with stroke often find themselves at a life stage where pivotal decisions regarding family and career are made [5]. In Australia, the cost implications of young stroke are well documented. In 2018–2019, the health system incurred an estimated $662.3 million in stroke-related expenses, with approximately $210.8 million attributed to the younger age group (under 65 years) [6].

There are unique age-related differences in the support and resources that people of different ages with stroke need. A study conducted by Keating and colleagues [7] underscored these differences, revealing that young adults under the age of 35 years with stroke were less inclined to seek help from healthcare professionals for their needs [7]. Moreover, a qualitative study focusing on the unmet needs of young adults revealed that stroke survivors under the age of 30 years expressed a preference for a distinct social support group separate from stroke survivors aged over 30 years [8]. These studies highlight the unique age-related differences in the support required post-stroke, emphasising the need for tailored strategies for different age groups.

Young stroke survivors face diverse challenges concerning their physical, emotional, communicative, cognitive, and psychosocial needs [7,8,9,10]. It is estimated that nearly 40% of individuals who have survived a stroke are discharged from medical facilities without any established provisions for their ongoing care needs [11]. One of the most frequently reported challenges by young stroke survivors is the difficulty in accessing resources tailored to their needs [7,8], limiting their ability to achieve optimal recovery and engagement in daily activities [12].

Technology has been employed to address the varied post-hospital discharge needs of stroke survivors [13,14]. Technology in this study encompasses a range of digital and interactive tools designed to support stroke rehabilitation that are delivered or accessed through common devices such as mobile phones, tablets, and computers and do not require external peripherals. Examples of these technologies are mobile apps, interactive games on mobile devices and tablets, and social media platforms. Mobile apps are utilised to assist clients with physical activity [15], provide stroke education [16], and send medication reminders via text messages [17]. Despite the wide use of technology to support stroke rehabilitation, there is a limited focus on how technology can specifically meet the unique needs of young adults. A recent study undertaken in Australia emphasises the potential role of technology in addressing the specific needs of young stroke survivors aged 18–30 while highlighting the challenges young stroke survivors face when accessing age-specific resources [8]. Recognising the pivotal role that technology plays in meeting the needs of young adults affected by stroke [8], it is important to note that the range of technological solutions available for young adults with stroke is not known and can be difficult to search for, posing accessibility challenges for community rehabilitation post-discharge from the hospital [18].

Developing a repository of resources could assist people going through the transition from care to the community, particularly benefiting those who experience fatigue after a stroke [8,19,20]. Additionally, knowledge of these resources could prove invaluable for healthcare professionals in supporting stroke survivors during their rehabilitation [21], thereby assisting in improving the quality of life and participation of young adults with stroke.

This study aimed to identify and collate the available technology designed to address the specific needs of young adults with stroke in Australia, with a view to developing a technological resource repository.

## 2. Materials and Methods

### 2.1. Study Design

An environmental scan was conducted to identify commercially available technologies that target the areas of need identified by young adults with stroke in Australia [22] (Table 1). No ethics approval was necessary as this study utilised information that was freely available in the public domain and there was no interaction with participants or retrieval of personal data.

The environmental scan methodology was selected due to its comprehensive approach, combining various data collection techniques to gain a holistic understanding of a topic’s internal and external contexts [23,24]. The environmental scan consisted of four phases: refinement (consultation), research, external review, and delivery [25].

### 2.2. Refinement Phase: Consultation Meetings

The environmental scan process followed steps similar to those in public health and health services research reported elsewhere [26]. To identify relevant information to guide the environmental scan, the authors consulted with the project’s advisory group, which consisted of 12 experts. This group included four young stroke survivors with at least three years of post-stroke experience, one speech and language specialist, three young stroke researchers, one neuropsychologist, one neurologist, one occupational therapist, and one social worker. All healthcare professionals in the group had over eight years of experience working in stroke care, and members were selected based on their extensive experience and significant contributions to the field. Meetings were conducted in November 2023 via Zoom teleconferencing software, and subsequent communication was via email. These consultations defined the scope of the scan, inclusion and exclusion criteria, sources of information, and keywords for searching. Following the meeting, a protocol was developed by the lead author (DA) and disseminated to the advisory members so they could provide their input.

### 2.3. Research Phase: Search for Resources

#### 2.3.1. Eligibility Criteria

The environmental scan’s inclusion criteria comprised the following: (1) an app or website that indicates “young stroke” with no age bracket; (2) apps designed for stroke survivors; (3) general reminder apps available in English; (4) both private and public social media platforms for young stroke survivors; (5) social media pages in Australia or other countries open to all young stroke survivors; (6) social media platforms set up by young stroke survivors; (7) and both free and paid apps.

Exclusion criteria comprised: (1) resources designed specifically for paediatric populations or populations over the age of 65 years; (2) general information not specific to stroke; (3) social media platforms with no information or description regarding the support group; (4) research publications; (5) social media platforms specified for stroke survivors from countries other than Australia; (6) social media platforms not for young stroke survivors; (7) and apps that can only be used in health facility settings.

#### 2.3.2. Data Sources and Searches

The environmental scan identified resources from a wide range of sources. This study included manual internet searches using the Google search engine, exploration of various websites, and a review of popular app stores such as the Google Play Store and Apple App Store. For the internet searches, a review of the first two pages of results was undertaken. Search engines were cleared between searches. Cookies were disabled before the search to avoid inadvertent bias. Additional data were collected via email and telephone from external sources such as rehabilitation centres across all Australian states, stroke organisations such as the Stroke Foundation of Australia, technology developers (Tactus Therapy-Vancouver, Canada, Flint Rehab-Irvine, CA, USA, Bungalowsoftware.com-USA), healthcare professionals (HCP), professional networks such as APA Neurology Physiotherapy Group, speech pathologists, physiotherapists, stroke nurses, exercise physiologists, and neurological rehabilitation groups based in Australia. These sources were recommended by the advisory group and deemed appropriate for gathering the necessary information to address the research objective. Non-respondents after two weeks were followed up with additional emails and telephone calls. Data collection spanned from December 2023–March 2024 and was undertaken by the lead author (DA). Search terms were discussed with the project’s advisory group which included individuals with lived experience. The aim was to include terms that reflect the variety of phrases stroke survivors might use when searching for apps related to exercises, speech rehabilitation, medication reminders, and peer support. A web or app store search was used to locate additional information on resources recommended by external sources. Two authors independently evaluated the identified resources to ensure they met the inclusion criteria and did not adhere to the exclusion criteria. Conflicts were resolved through discussions with an additional panel of four authors (MLB, SP, CM, MS). Definitions of the terms used in this article are outlined in Table 2, while Table 3 details the search strategy.

#### 2.3.3. Data Extraction

One author (DA) independently extracted information on eligible apps and social media platforms for young stroke survivors. The information included resources shared by external sources such as healthcare professionals, organisations, rehabilitation centres, and technology developers. The data were collected using a standardised Microsoft Excel spreadsheet. The data were validated by two other authors (MS, SP). The data collected included, but were not limited to, the name of the app or social media platform; characteristics of the source, including the URL; basic description/features; operating system; cost; language(s) available; target audience; the year of the last update or review; and the name of the developer. The authors piloted the extraction sheet on the first five resources under each category to reconcile any differences. The data extraction sheet was revised to include additional details about app descriptions. Following these modifications, the pilot data were updated and retained.

### 2.4. External Review Phase

The collated resources were reviewed by the project’s advisory group. The aim was to identify possible ways to categorise the data to address the specific needs of young stroke survivors.

### 2.5. Delivery Phase

The list of resources arising from this environmental scan will be publicly available via a national digital platform and as a Supplemental File to this manuscript (Tables S1–S4).

### 2.6. Data Categorisation

The extracted data were categorised and visually represented in tables and figures. The categorisation process was iterative, involving discussions with team members to determine the most effective approach, as seen in a similar study [25]. The collated resources were assessed based on three main categories: independent use (apps that can be utilised without the assistance of a healthcare professional), cost effectiveness (free apps and apps with free basic functions along with in-app purchases options, versus paid apps), and whether it was updated less than 1 year ago. This categorisation was informed by a previous young stroke study, where participants expressed a preference for technological resources that are contemporary, cost effective, and supportive of independence [8]. These categories were compared across all available platforms because not all apps are available on all the platforms. By categorising apps according to these platforms, users can conveniently find apps that are compatible with their devices (Table S5).

## 3. Results

The environmental scan identified 114 resources which included interactive games for re-training limb movement (n = 12, 11%), speech rehabilitation apps (n = 46, 40%), medication reminder apps (n = 23, 20%), and social media platforms offering peer mentoring and support (n = 33, 29%).

Thirty two of the 76 sources who were contacted via email and telephone, including healthcare professionals/networks, rehabilitation centres, stroke organisations, and technology developers, responded. Among these, 14 healthcare professionals were included: nine physiotherapists, one speech and language therapist, one exercise physiologist, and three stroke nurses. Ten responses were received from rehabilitation centres and hospitals without attribution to a specific healthcare professional. The remaining responses were from professional networks, technology developers, and the advisory group members.

### 3.1. Characteristics of Apps

In total, 81 apps were identified for re-training movement of limbs, speech rehabilitation, and medication reminders. Most apps can be used independently, without the assistance of a healthcare professional (n = 74, 91%). More than half (n = 51, 63%) were updated within the last year, while some (n = 10, 12%) had their last update more than a year ago, and the update time was not specified for others (n = 20, 25%). More than half of the apps (n = 42, 52%) were paid, 38 (47%) were free of charge, and 1 offered both paid and free options. All the apps were available in English, with some offering support for multiple languages. The apps were accessible on various platforms, with 32 (40%) available on both app stores, followed by websites (n = 22, 27%), Apple App Store only (n = 19, 23%), and a few on the Google Play Store only (n = 8, 10%). A few of the apps (n = 3, 4%) had the same developer and shared identical features, yet were listed under different names on the Apple App Store and Google Play Store (see Table 4).

Most interactive games for speech rehabilitation were user-paid (n = 36, 78%). All of the medication reminder apps targeted the general population. Most of these apps were developed with input from healthcare professionals (n = 45, 56%) or based on users/published evidence (n = 13, 16%), but for 23 (28%) this aspect was unspecified.

### 3.2. Characteristics of Social Media Posts

Of the 33 social media posts identified, Facebook accounted for the majority (n = 21, 64%), followed by Instagram (n = 7, 21%), TikTok (n = 3, 9%), YouTube (n = 1, 3%), and other websites (n = 1, 3%). Most social media posts were publicly available (n = 26, 79%), with some (n = 7, 21%) being private. A total of 15 young stroke social media posts did not specify a target age group (46%), with 12 on Facebook. All TikTok, Instagram, and YouTube young stroke posts did not indicate the target population. In all, 61 (8%) of the social media platforms had a broad target age group (<65 years, <55 years, 18–65 years) followed by an age group between 15 and 45 years (n = 3, 19%). The purpose of these support groups is multifaceted, including providing emotional support, sharing inspiring experiences of the stroke journey, organising social catchups, creating awareness, sharing resources, and celebrating achievements.

### 3.3. Distribution of Apps Categorised by Various Platforms

**Figure 1:**
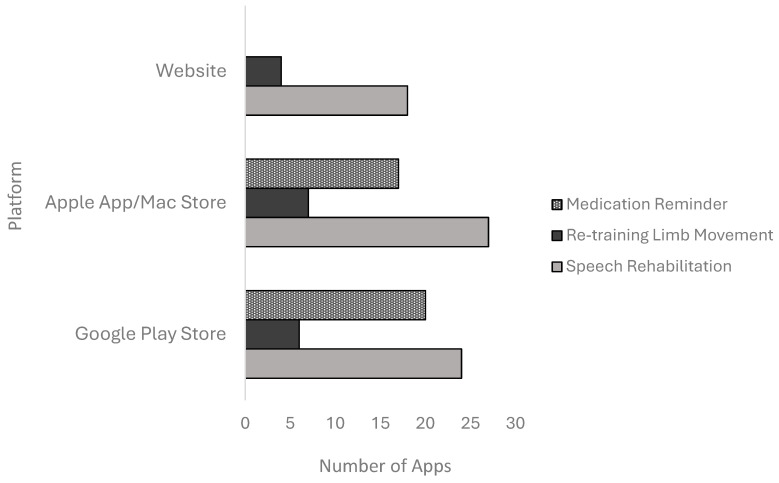
Distribution of the number of apps across various platforms.

Interactive game apps for speech rehabilitation (n = 23, 50%) and re-training movement of limbs (n = 5, 42%) were available on both the Apple App Store and the Google Play Store, while medication reminder apps were more predominant on the Apple App Store only (n = 13, 57%). There were no medication reminder apps available on the web (Figure 1).

### 3.4. Categorisation of Social Media Platforms Based on Target Age Group

**Figure 2:**
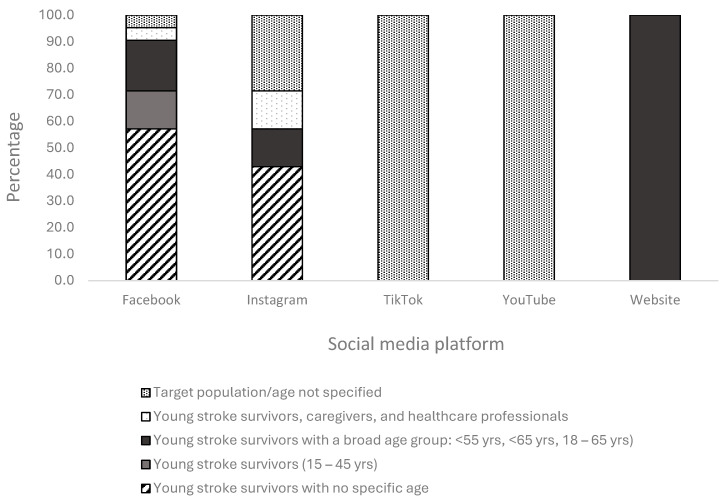
Percentage of target age group by type of social media platform.

The social media platforms identified were categorised based on target audience: young stroke survivors with no specified age, young stroke survivors with a broad age group (<55 years, <65 years, 18–65 years), young stroke survivors (15–45 years), young stroke survivors with carers/healthcare professionals, and those with no target population specified (Figure 2).

## 4. Discussion

This environmental scan aimed to identify and collate available technological resources that address the specific needs of young adults with stroke in Australia, with the intention to provide access to these resources hosted by a national digital stroke platform. This environmental scan has led to the development of a comprehensive repository of interactive games for re-training movement of limbs, speech rehabilitation and medication reminder apps, and various social media platforms, which represent a promising approach to addressing the challenges young stroke survivors face in accessing rehabilitation resources.

This research found that most of these apps can be used independently without healthcare professional involvement. This finding highlights the increasing autonomy and self-management capabilities these apps provide to users. However, a considerable proportion of these apps, particularly those focused on speech rehabilitation, are user-paid. This finding is a major accessibility barrier as 25–50% of stroke survivors do not return to work after a stroke [27,28,29], thus resulting in a potential for high financial burden [30]. Moreover, considering that an estimated one-third of people with stroke will be diagnosed with aphasia or have communication difficulties, coupled with stroke’s effects of social isolation, depression, and lower quality of life [31,32,33], this study suggests the need to find a way to make these apps freely accessible, to assist in addressing the unique needs of young stroke survivors. This finding aligns with a recent systematic review of mobile apps for stroke survivors and caregivers in the US Apple iTunes store, which revealed that more than half of the apps were paid [34]. In contrast, a recent environmental scan on mHealth apps for dementia and Alzheimer’s disease found that most apps were free [35]. This discrepancy underscores a gap in the development and distribution of stroke rehabilitation apps. Ensuring that these apps are freely accessible could reduce the financial burden on stroke survivors and also contribute to enhancing their quality of life and participation, particularly in young adults, who may face a long-term recovery journey.

Lack of motivation is one of the barriers to physical activity, which interactive games may address [36]. Evidence suggests that games are more motivating and fun than traditional interventions [37] and can have positive health-related benefits [37,38]. Nonetheless, this study found a small number of interactive games available to assist with improving limb function for stroke survivors. While there is limited environmental scan literature specifically on technological resources for young stroke survivors, our findings align with the systematic reviews by Piran et al. [34] and Cao et al. [39], who noted that the majority of existing apps for stroke survivors and caregivers primarily focus on language and communication difficulties, with few addressing physical rehabilitation. Given these findings, there is a clear need to develop more interactive games to assist young stroke survivors in improving their limb function at home post-discharge. This assistance could potentially enhance their recovery by increasing motivation and engagement in physical activity.

The scan highlights potential barriers young stroke survivors might face when accessing social media platforms, especially in terms of finding age-specific resources and selecting the right keywords for app searches. The findings reveal that while the young stroke social media platforms identified offer diverse support, including sharing personal experiences, assisting with challenges, and raising awareness about stroke symptoms and prevention, many do not specify the target age group category. A recent review of mobile apps available on iOS and Google Play Store for stroke rehabilitation revealed that approximately 81% of the apps did not specify the intended age group, which is consistent with our findings [39]. This finding could account for the possible reasons why young adults with stroke find it challenging to access age-specific resources. Moreover, considering the heterogeneity in the definition of young stroke globally ranging from 18–65 years [7,10,40,41,42], without a specific targeted age group, it may be difficult to address exact needs of individuals. This assertion is corroborated by a recent study conducted by Amoah et al. [8], which reported feelings of isolation among young stroke survivors under 30 years of age despite their participation in a young stroke support group composed of individuals over 30 years old. Consequently, this study recommends that social media platforms designed for young stroke survivors should consider clearly indicating the target age group, to harness the advantages of peer support, such as emotional, physical, and psychological support [43], and improve engagement in self-management [44]. Additionally, the scan underscores the importance of the choice of keywords and punctuation in finding the resources available through popular app stores, and it reveals a small number of apps that have identical features and descriptions, as well as the same developer, but are listed with different names on different platforms [45]. This inconsistency in naming could potentially lead to confusion among stroke survivors, particularly those who are not technologically inclined or have a high level of cognitive impairment and might be searching for these apps for their rehabilitation needs. It is suggested that stroke survivors pay particular attention to the keywords and punctuation used when searching for these apps, to prevent confusion.

### Strengths and Limitations

Strengths of this study include expert and end-user consultation and a search of a wide range of sources to establish a repository of resources for young stroke survivors. Additionally, no previous studies have scanned the environment of apps and social media platforms available to meet the specific needs of young adults with stroke in Australia.

While the environmental scan provides valuable insights into the landscape of apps available for young stroke survivors, it is essential to acknowledge its limitations. The determination of whether the apps could be used independently without a healthcare professional was based solely on developer websites and app descriptions, which may not always provide accurate information. Further research assessing the usability of these apps is necessary to have a clearer understanding. Additionally, due to the dynamic nature of internet content, it is impossible to guarantee that all relevant and currently available apps will remain accessible. To address this limitation, collaborating with a national stroke organisation in Australia to host and regularly update these collated resources would help to ensure their currency. Furthermore, this scan did not assess the effectiveness or user satisfaction of these apps. Future studies should involve young stroke survivors in evaluating the quality and usability of these apps to provide a more comprehensive understanding of their impact.

## 5. Conclusions

This environmental scan provides valuable insights into the current technology-based tools available to meet young stroke survivors’ needs regarding re-training movement of the limbs, speech rehabilitation, medication reminders, and peer support. While many apps are actively maintained and can be used independently, there are still accessibility issues and potential barriers to young stroke survivors finding these resources. The collation of information to direct young adults with stroke and their healthcare professionals to available technological resources offers a practical solution that can enhance rehabilitation and transition to ongoing care.

## Figures and Tables

**Table 1:** Technology-based tools for specific needs of young adults with stroke [22].

	Technology-Based Tool	Area of Need
1	Interactive games	Train and re-train movement of limbs
		Speech rehabilitation and communication
2	Apps	Reminder for medications
3	Social media	Mentorship of newly diagnosed stroke survivors by experienced survivors for support

**Table 2:** Definitions for the terms used in the article.

No	Term	Definition
1	Interactive games	Activity-based games that require participation and decision making within the game environment. Activity-based apps that require participation and decision making within the game environment.
2	Apps (applications)	Software programs on electronic devices such as mobile telephones, websites, smartwatches, tablets, and computers.
3	Social media	A technology-based platform where ideas and information are shared among online communities and networks. This term could include Facebook, X, Instagram, TikTok, and YouTube.
4	Re-train movement of limbs	Improving control and coordination of the limbs, such as functional movement of the arms or legs.
5	Speech rehabilitation	Improving speech production, language skills, coordination of speech, and social communication skills. This term could include resources for reading, listening, comprehension, understanding, speaking, or writing.
6	Mentorship for young stroke survivors	A person or group of people that provide understanding, guidance, and lived experience to support a young person living with stroke. This term could include sharing stories, sharing resources, or peer support.
7	Medication reminder	A reminder to take medication, or to collect or obtain new prescriptions for medication.

**Table 3:** Search parameters (Google Play Store, Apple App Store, websites).

Area	Search Sources	Keywords
Train and re-train movement of limbs	Google Play Store, Apple App Store	Movement, re-train, post-stroke exercise app, limb exercises, stroke exercises
	Websites	
Speech rehabilitation and communication	Google Play Store, Apple App Store	Aphasia, language, speech disorder, speech therapy, speech pathology, voice swallowing, speech therapy apps, speech rehabilitation, communication support
	Websites	
Reminder for medications	Google Play Store, Apple App Store	Medication reminders, medication adherence, medication compliance, pill reminders
	Websites	
Mentorship of newly diagnosed stroke survivors by experienced survivors for support	Social media platforms (Facebook, TikTok, YouTube, Instagram, X)	Stroke, social network, social support group, young stroke survivors, peer support, peer mentoring

**Table 4:** Comparison of identical apps with different names on Apple App Store and Google Play Store.

Purpose of App	Name on Apple App Store	Name on Google Play Store	Developer
Speech rehabilitation	Language Therapy 4-in-1	Language Therapy: Aphasia	Tactus
	Category therapy	Category therapy: categories	Tactus
Medication reminder	Dosecast: My pill Reminder app	Dosecast—Pill Reminder & Med	Montuno Software, LLC

## Data Availability

All data generated or analysed during this study are included in this published article.

## Supplementary Materials

The following supporting information can be downloaded at: https://www.mdpi.com/article/10.3390/ijerph21091254/s1, Table S1: Interactive games for speech rehabilitation; Table S2: Interactive games for re-training limb movement; Table S3: Medication reminder apps; Table S4: Social media platforms for peer mentoring and support; Table S5: Repository of resources.

## References

[1] Murray C.J., Aravkin A.Y., Zheng P., Abbafati C., Abbas K.M., Abbasi-Kangevari M., Abd-Allah F., Abdelalim A., Abdollahi M., Abdollahpour I. (2020). Global burden of 87 risk factors in 204 countries and territories, 1990–2019: A systematic analysis for the Global Burden of Disease Study 2019. Lancet.

[2] Vos T., Lim S.S., Abbafati C., Abbas K.M., Abbasi M., Abbasifard M., Abbasi-Kangevari M., Abbastabar H., Abd-Allah F., Abdelalim A. (2020). Global burden of 369 diseases and injuries in 204 countries and territories, 1990–2019: A systematic analysis for the Global Burden of Disease Study 2019. Lancet.

[3] Feigin V.L., Stark B.A., Johnson C.O., Roth G.A., Bisignano C., Abady G.G., Abbasifard M., Abbasi-Kangevari M., Abd-Allah F., Abedi V. (2021). Global, regional, and national burden of stroke and its risk factors, 1990–2019: A systematic analysis for the Global Burden of Disease Study 2019. Lancet Neurol..

[4] Deloitte Access Economics (2020). No Postcode Untouched, Stroke in Australia 2020.

[5] Walters R., Collier J.M., Carvalho L.B., Langhorne P., Katijjahbe M.A., Tan D., Moodie M., Bernhardt J. (2020). Exploring post acute rehabilitation service use and outcomes for working age stroke survivors (≤65 years) in Australia, UK and South East Asia: Data from the international AVERT trial. BMJ Open.

[6] Australia Institute of Health and Welfare A. Disease expenditure in Australia 2018–2019. https://www.aihw.gov.au/reports/health-welfare-expenditure/disease-expenditure-australia/data.

[7] Keating J., Borschmann K., Johns H., Churilov L., Bernhardt J. (2021). Young Stroke Survivors’ Preferred Methods of Meeting Their Unique Needs: Shaping Better Care. Neurology.

[8] Amoah D., Prior S., Mather C., Schmidt M., Bird M.-L. (2023). Exploring the Unmet Needs of Young Adults with Stroke in Australia: Can Technology Help Meet Their Needs? A Qualitative Study. Int. J. Environ. Res. Public Health.

[9] Abrahamson V., Wilson P.M. (2019). How unmet are unmet needs post-stroke? A policy analysis of the six-month review. BMC Health Serv. Res..

[10] Shipley J., Luker J., Thijs V., Bernhardt J. (2020). How can stroke care be improved for younger service users? A qualitative study on the unmet needs of younger adults in inpatient and outpatient stroke care in Australia. Disabil. Rehabil..

[11] Prvu Bettger J., McCoy L., Smith E.E., Fonarow G.C., Schwamm L.H., Peterson E.D. (2015). Contemporary trends and predictors of postacute service use and routine discharge home after stroke. J. Am. Heart Assoc..

[12] Rakesh N., Boiarsky D., Athar A., Hinds S., Stein J. (2019). Post-stroke rehabilitation: Factors predicting discharge to acute versus subacute rehabilitation facilities. Medicine.

[13] Langan J., DeLave K., Phillips L., Pangilinan P., Brown S.H. (2013). Home-based telerehabilitation shows improved upper limb function in adults with chronic stroke: A pilot study. J. Rehabil. Med..

[14] Chen J., Jin W., Dong W.S., Jin Y., Qiao F.L., Zhou Y.F., Ren C.C. (2017). Effects of home-based telesupervising rehabilitation on physical function for stroke survivors with hemiplegia: A randomized controlled trial. Am. J. Phys. Med. Rehabil..

[15] Krishnamurthi R., Hale L., Barker-Collo S., Theadom A., Bhattacharjee R., George A., Arroll B., Ranta A., Waters D., Wilson D. (2019). Mobile technology for primary stroke prevention: A proof-of-concept pilot randomized controlled trial. Stroke.

[16] Kang Y.-N., Shen H.-N., Lin C.-Y., Elwyn G., Huang S.-C., Wu T.-F., Hou W.-H. (2019). Does a Mobile app improve patients’ knowledge of stroke risk factors and health-related quality of life in patients with stroke? A randomized controlled trial. BMC Med. Inform. Decis. Mak..

[17] Zeng Z., Wu T., Lv M., Qian J., Chen M., Fang Z., Jiang S., Zhang J. (2022). Impact of mobile health and telehealth technology on medication adherence of stroke patients: A systematic review and meta-analysis of randomized controlled trials. Int. J. Clin. Pharm..

[18] Gopaul U., Charalambous M., Thilarajah S., Kwah L.K., Chapman S., Bayley M., Demers M. (2022). Age-specific information resources to address the needs of young people with stroke: A scoping review protocol. Syst. Rev..

[19] Cumming T.B., Packer M., Kramer S.F., English C. (2016). The prevalence of fatigue after stroke: A systematic review and meta-analysis. Int. J. Stroke.

[20] Paciaroni M., Acciarresi M. (2019). Poststroke Fatigue. Stroke.

[21] Tulek Z., Poulsen I., Gillis K., Jönsson A.C. (2018). Nursing care for stroke patients: A survey of current practice in 11 European countries. J. Clin. Nurs..

[22] Amoah D., Prior S., Mather C., Schmidt M., Bird M.-L. (2024). Preferred Technology-Based Tools for Meeting the Needs of Young Adults with Stroke in Australia: A Cross-Sectional Survey. Health Technol..

[23] Rowel R., Moore N.D., Nowrojee S., Memiah P., Bronner Y. (2005). The utility of the environmental scan for public health practice: Lessons from an urban program to increase cancer screening. J. Natl. Med. Assoc..

[24] Conway M. (2012). Doing environmental scanning: An overview guide. Think. Futures Hotham Hill. Retrieved August.

[25] Flowers E., Saha S., Allum L., Rose L. (2024). An environmental scan of online resources for informal family caregivers of ICU survivors. J. Crit. Care.

[26] Wilburn A., Vanderpool R.C., Knight J.R. (2016). Peer reviewed: Environmental scanning as a public health tool: Kentucky’s human papillomavirus vaccination project. Prev. Chronic Dis..

[27] Glader E.L., Jonsson B., Norrving B., Eriksson M. (2017). Socioeconomic factors’ effect on return to work after first stroke. Acta Neurol. Scand..

[28] Larsen L.P., Biering K., Johnsen S.P., Andersen G., Hjollund N.H. (2016). Self-rated health and return to work after first-time stroke. J. Rehabil. Med..

[29] Turi E.R., Conley Y., Crago E., Sherwood P., Poloyac S.M., Ren D., Stanfill A.G. (2019). Psychosocial comorbidities related to return to work rates following aneurysmal subarachnoid hemorrhage. J. Occup. Rehabil..

[30] Maaijwee N.A., Rutten-Jacobs L.C., Schaapsmeerders P., Van Dijk E.J., de Leeuw F.-E. (2014). Ischaemic stroke in young adults: Risk factors and long-term consequences. Nat. Rev. Neurol..

[31] Baker C., Worrall L., Rose M., Hudson K., Ryan B., O’Byrne L. (2018). A systematic review of rehabilitation interventions to prevent and treat depression in post-stroke aphasia. Disabil. Rehabil..

[32] Sjöqvist Nätterlund B. (2010). A new life with aphasia: Everyday activities and social support. Scand. J. Occup. Ther..

[33] Hilari K. (2011). The impact of stroke: Are people with aphasia different to those without?. Disabil. Rehabil..

[34] Piran P., Thomas J., Kunnakkat S., Pandey A., Gilles N., Weingast S., Burton D., Balucani C., Levine S.R. (2019). Medical mobile applications for stroke survivors and caregivers. J. Stroke Cerebrovasc. Dis..

[35] Ali S., Alizai H., Hagos D.J., Rubio S.R., Calabia D., Serrano Jimenez P., Senthil V.A., Appel L. (2024). mHealth Apps for Dementia, Alzheimer Disease, and Other Neurocognitive Disorders: Systematic Search and Environmental Scan. JMIR mHealth uHealth.

[36] Poltawski L., Boddy K., Forster A., Goodwin V.A., Pavey A.C., Dean S. (2015). Motivators for uptake and maintenance of exercise: Perceptions of long-term stroke survivors and implications for design of exercise programmes. Disabil. Rehabil..

[37] Johnson D., Deterding S., Kuhn K.-A., Staneva A., Stoyanov S., Hides L. (2016). Gamification for health and wellbeing: A systematic review of the literature. Internet Interv..

[38] Sardi L., Idri A., Fernández-Alemán J.L. (2017). A systematic review of gamification in e-Health. J. Biomed. Inform..

[39] Cao W., Kadir A.A., Wang Y., Wang J., Dai B., Zheng Y., Mu P., Hu C., Chen J., Na L. (2023). Description of apps targeting stroke patients: A review of apps store. Digit. Health.

[40] O’Hana S.N.C. (2021). Preferences of Young Stroke Survivors to Meet Their Unique Needs: It Is Time to Listen. Neurology.

[41] Opoku S., Eliason C., Akpalu A. (2020). Why me?: A qualitative study on the experiences of young stroke survivors in the Accra Metropolis of Ghana, West Africa. J. Patient Exp..

[42] Harris Walker G., Oyesanya T.O., Hurley A., Sandhu S., Liu C., Mulla M., Prvu Bettger J. (2021). Recovery experiences of younger stroke survivors who are parents: A qualitative content analysis. J. Clin. Nurs..

[43] Wan X., Chau J.P.C., Mou H., Liu X. (2021). Effects of peer support interventions on physical and psychosocial outcomes among stroke survivors: A systematic review and meta-analysis. Int. J. Nurs. Stud..

[44] Clark E., Maccrosain A., Ward N.S., Jones F. (2020). The key features and role of peer support within group self-management interventions for stroke? A systematic review. Disabil. Rehabil..

[45] Alharbi K., Blackshear S., Kowalczyk E., Memon A.M., Chang B.-Y.E., Yeh T. (2014). Android Apps Consistency Scrutinized.

